# Investigating the Influencing Factors and Correlation Between Popularity and Emotion of Public Opinion During Disasters: Evidence from the “7.20” Rainstorm in China

**DOI:** 10.3390/bs15020176

**Published:** 2025-02-07

**Authors:** Anying Chen, Yixuan Liu, Yanlin Huang, Guofeng Su, Dinghuan Yuan

**Affiliations:** 1 School of Public Administration and Emergency Management, Jinan University, Guangzhou 510632, China; chenay@jnu.edu.cn (A.C.); yixuan@stu2023.jnu.edu.cn (Y.L.); yanlin_elynn@163.com (Y.H.); 2 Public Policy Institute, Jinan University, Guangzhou 510632, China; 3 Institute of Public Safety Research, Department of Engineering Physics, Tsinghua University, Beijing 100084, China; sugf@tsinghua.edu.cn

**Keywords:** public opinion, disasters, online social networks, case study

## Abstract

Disasters not only directly cause casualties and property losses but also significantly impact public opinion. In order to identify the evolution characteristics and influencing factors of public opinion during disasters, this paper put forward an analytical framework for analyzing public opinion, which clarified the relationships among key characteristics of public opinion and emphasized the role of official agencies in the processes of information releasing and information feedback. Guided by this framework, this paper collected the public opinion on Sina Weibo during the extraordinary “7.20” rainstorm in Henan Province, China. By analyzing the changes in the discussion regarding both the popularity of and the emotion displayed in Sina Weibo comments over time, it was found that the evolution of public opinion was closely related to disaster development. Novel informational content or innovative forms of information contribute to enhancing the discussion of popularity, while the events or emotions expressed within the information elicit corresponding emotional responses from the public. As popularity increased, the prevalence of negative emotions was observed to diminish concurrently with a rise in the proportion of neutral emotions. Based on these results, some suggestions on the management of public opinion during disasters were put forward.

## 1. Introduction

Natural hazards can have a significant influence on industrial and agricultural production, transportation, communications, the ecological environment, and people’s daily lives. As a result, both the government and the public have a negative perception when facing a critical situation caused by natural hazards, which could easily cause a crisis of public opinion. The expression of online public opinion easily exceeds the progression of rational expression, and dormant social contradictions will be intensified. This also triggers the dissemination of rumors, Internet storms, hot spots, and other forms of chaos, disrupting the release and dissemination of useful messages and amplifying citizens’ doubts and dissatisfaction regarding the ability of local governments to govern. Thus, the management of public opinion during disasters has become a hot issue for governments, necessitating deep investigation into the patterns and underlying causes that give rise to it.

Thanks to the rapid development of data mining and analysis, it has become possible to discover the language characteristics of the words expressed by the public. In general, previous research usually analyzed public opinion by considering aspects such message popularity and public emotion, which are the two main indicators of negative public opinion events (T. Li et al., 2022; Meng et al., 2022; C. Yang, 2022). On one hand, it is generally recognized that the popularity of crisis information is closely in accordance with the development of the corresponding events, which has been verified by cases such as the COVID-19 epidemic, Hurricane Sandy, the Louisiana flood, and Storm Cindy (Han et al., 2020; Kim et al., 2018; Kim & Hastak, 2018). On the other hand, the public emotions displayed on online social networks for different emergencies have also been a hot spot in the area of communication and psychology. The advancement of natural language processing (NLP) technologies has spurred the development of numerous novel methods for identifying the sentiment polarity and emotional categories of text on online social networks (Raad et al., 2018). Through the methods of machine learning and sentiment dictionary, it was found that different kinds of public emotions expressed on online social networks during crisis events shared a consistent temporal trend that grew rapidly in a short time and then gradually fell until they disappeared, meaning that the initial stage of the emergency was the best timing for the government to control public opinion (S. Li et al., 2020). However, some research also found that negative emotions decreased and positive emotions increased throughout the entire emergency, which appears to be inconsistent with the above findings (Ukkusuri et al., 2014; Zhao et al., 2020).

Many studies have conducted a multifaceted analysis of the factors that influence information popularity and public sentiment on social media. These investigations have explored the impact of various elements, including user types, thematic content, hashtags, media formats, users’ locations, and both the form and content of information, on the popularity and emotion of posts (Fan et al., 2020; Kankanamge et al., 2020). Additionally, these studies have delved into the complex interplay between social media usage, psychological well-being, and the formation of public opinion (Ferrara & Yang, 2015; Winter & Neubaum, 2016; Woods, 2023; Yue et al., 2019). We can categorize the message-related elements among these influential factors into two categories: message content and message form. Empirical studies have historically demonstrated that these two elements are significant factors that influence information popularity and public emotion. While it is acknowledged that the content and form of messages are interrelated, the mechanisms by which the two factors influence information popularity and public emotions remain to be fully clarified. An in-depth investigation of this question is crucial for developing a comprehensive framework that can predict and potentially guide public opinion on social media platforms in a positive direction during disasters.

Furthermore, while some studies have explored the interplay between information popularity and public emotions within their analytical processes or results (Lu & Hong, 2022; Qu et al., 2011; X. Wang et al., 2018), how they connect with each other still remains confusing. Typically, these two elements are employed to depict the state of public opinion, yet there is a lack of clarity on how to portray public emotion and how these factors contribute to instances of adverse public opinion events. A theoretical framework explaining the relationship between information popularity and public emotion and the representation of public opinion in the context of negative opinion events is needed.

Inspired by the above literature, this paper attempts to explore the following three research questions: What are the driving forces behind public opinion events, particularly in terms of topic popularity and public emotion? What is the relationship between information popularity and public emotion? And how can the development of negative public opinion events be effectively improved or intervened upon? To address these questions, first, an analytical framework for analyzing online public opinion is put forward. Second, guided by the framework, the temporal characteristics of public opinion, including topics of concern, topic popularity, and people’s emotions are investigated. Third, the relationship between micro-blog popularity and people’s emotion is analyzed. Fourth, the influence of the micro-blogs’ content and form on the performance of public opinion is studied. Finally, some suggestions for public opinion governance in the context of disasters based on the research findings are put forward.

## 2. Methodology

### 2.1. Analytical Framework

When disasters happen, media agencies are responsible for releasing related information. According to White’s gatekeeper theory (White, 1950), these agencies can also be regarded as gatekeepers because their process of filtering information directly determines what the public can read. In other words, how these agencies convey the information could change the public’s response. When the public receives the filtered information, they react by posting micro-blogs or comments on online social media, forming public opinion on disasters.

In current studies, the development of public opinion is mainly reflected by the characteristics of discussion topics, popularity, and emotional tendency. It is generally accepted that emotionally charged messages spread more widely (X. Wang et al., 2018). Although the question of whether positive or negative messages spread more widely has not reached an agreed conclusion (Ferrara & Yang, 2015; Thelwall et al., 2011), it is clear that topic popularity and public emotion develop simultaneously (M. Cai et al., 2021). The public’s concern for a particular issue can influence their emotional response, which, in turn, can regulate the interaction between different issues when they compete for public attention (Peng et al., 2017). As for the discussion topic factor, it has often been used to explain the changes in discussion popularity and public emotion. For example, Su et al. and Pang et al. used the discussion keywords to explain public sentiment evolution at different stages (Pang et al., 2020; Su et al., 2021), and Zhao et al. used hot topic keywords to explain the changing trend of the public’s attention with respect to a specific issue (Ferrara & Yang, 2015; X. Wang et al., 2018; Zhao et al., 2020). Similar to Schramm’s mass communication model (Schram, 1954), these three factors act as the feedback in the information releasing process, which keeps relevant agencies acquainted with the development of public opinion. It helps the agencies adjust their information release strategy in time so as to prevent negative public opinion events (Tatar et al., 2014; Zhou & Zhang, 2017).

**Figure 1:**
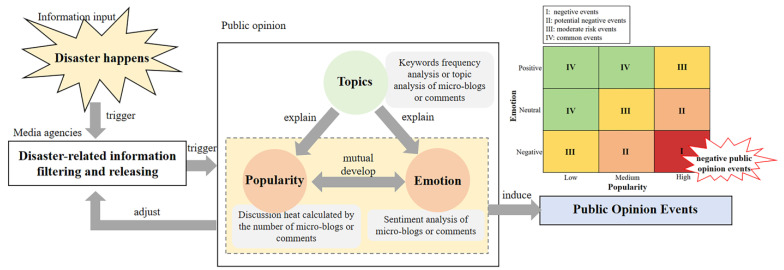
The public opinion analysis framework.

Based on the above findings, an analytical framework for analyzing online public opinion is summarized in Figure 1. When disasters happen, the media agencies filter and release disaster-related information to the public, causing the public to express their opinions, which are shaped by three characteristics, namely, discussion topics, popularity, and emotion. Here, the public’s topics of concern are usually acquired through the methods of keywords frequency analysis or topic analysis. Topic popularity refers to the discussion’s heat and is usually calculated via the number of topic-related micro-blogs or public comments. As for the public’s emotions, these are the emotions expressed in micro-blogs or comments, which can be calculated via sentiment dictionary methods or machine learning. According to previous research, the two factors of popularity and emotion influence each other and develop together, and the topic factor acts as the explanation of the former two factors during the analysis of public opinion. By monitoring the development of these three factors, media agencies can learn the current state of public opinion in time and adjust their subsequent information release strategy accordingly.

What is more, the factors of popularity and emotion can be indicators of different public opinion events (Z. Cai et al., 2021; Y. Li, 2022). Referring to the risk matrix in the field of risk evaluation, we use a matrix to evaluate public opinion according to the factors of popularity and emotion. Four public opinion event levels were identified: Level I indicates negative public opinion events, with the characteristics of high popularity and negative emotion, which should be avoided if possible. Level II represents events with medium popularity and negative emotion or events with high popularity and neutral emotion. These events are potential negative public opinion events and should be paid close attention. Level III and Level IV indicate moderate- and low-risk public opinion events, respectively, which are not likely to provoke unexpected public opinion events.

In all, this framework elaborates on the processes of public opinion management and clarifies the relationships among the three indicators of public opinion events. An evaluating method of public opinion events according to topic popularity and public emotion is also integrated into it. Furthermore, it emphasizes the effect of relevant agencies’ information release strategy on the development of public opinion, highlighting the significance of public opinion monitoring and management. It could serve as a guidance framework for public opinion analysis and is applied in the following case study.

### 2.2. Event Description

This paper selected the 7.20 rainstorm in Henan Province, China, in 2021 as its case study. At first, the rainfall was relatively weak and concentrated in northern Henan on 17 July. From 19 July, the rainfall transferred southward to Zhengzhou and consistently strengthened to 201.9 mm per hour, which broke the historical maximum hourly precipitation record in mainland China. Later, on 22 July, the rainfall center transferred northward again. During these days, the Meteorological Bureau of Henan Province issued the highest level of rainstorm warning several times. The rainstorm gradually decreased and ended on 23 July.

The intense rainstorm caused severe urban flooding, and the water level reached as high as the waists of pedestrians in low-lying areas of Zhengzhou in a short time on 20 July, immensely interrupting the social and economic normal order. An incident that should be mentioned was that over 400 people were trapped in a metro carriage when the rainwater poured into the metro station, which eventually led to 14 people’s deaths. This event quickly attracted a high number of negative comments from online users across the whole of the Internet in China, triggering a serious crisis of public opinion. The large-scale and high-intensity nature of the discussion during this rainstorm made it an appropriate case study from which to learn about public opinion crises.

### 2.3. Data Collection

In order to learn about the characteristics of public opinion during severe disasters, this paper collected a series of related comments from users of Sina Weibo. According to the preliminary investigation, the official account of People’s Daily issued the highest number of micro-blogs about the rainstorm in Henan Province and obtained the most comments from online users among all the official accounts of government bodies and news media. Therefore, the micro-blogs regarding the heavy rainstorm in Henan issued by People’s Daily were selected, and all the popular comments on these micro-blogs were collected using the web crawling method. Finally, a total of 146 micro-blogs issued by People’s Daily and 94,637 comments on these selected micro-blogs were collected.

### 2.4. Data Analysis Methods

The collected data were first pre-processed by deleting items with duplicate contents and comments with missing information, segmenting the sentences into words, and deleting the stop-words among the results of the word segmentation. Then, sentiment analysis and topic analysis were conducted with these processed data. In this paper, the public’s most popular topics were measured according to the word frequency of the comments under the selected micro-blogs. A sentiment analysis was conducted using the sentiment dictionary method, which calculated the sentiment scores for sentences based on the sentiment score for each word set in the sentiment dictionary. We used the sentiment dictionary from Gooseeker, an example of big data processing software, to determine the emotional tendency of the comments about the rainstorm of Henan Province on Sina Weibo.

## 3. Results

### 3.1. Temporal Changes of Public Opinion on the Rainstorm

According to the analytical framework, the temporal changes of the rainstorm-related micro-blog popularity and the public’s emotion, which were the two fundamental indicators of public opinion, should be studied. And in order to further learn about the causes of the temporal changes of popularity and the public’s emotion, the fundamental characteristics of the public’s topics of concern should be analyzed first.

#### 3.1.1. The Public’s Topics of Concern

**Figure 2:**
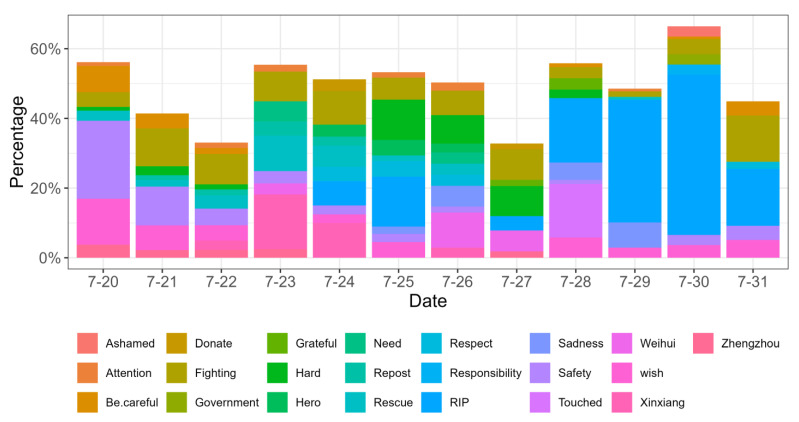
The daily keywords of the public’s comments during the rainstorm.

The public’s topics of concern during the rainstorm were analyzed by calculating the frequency of certain keywords, and the top ten keywords and their relative proportion for each day during the rainstorm are shown in Figure 2.

In general, the temporal changes of these keywords were roughly consistent with the development of the rainstorm disaster. On 20 July, when the rainstorm suddenly broke out in the city of Zhengzhou, the public was more likely to express their concern, as well as wishes and encouragement for the victims’ safety, resulting in the keywords “safety”, “wish”, “be careful”, and “fighting” ranking as the top four over the following two days. After that, the frequency of keyword “rescue” increased continuously until 25 July, indicating that people diverted their attention to the actual rescue progress. At the same time, the ranking of “Zhengzhou” declined while “Xinxiang” and “Weihui” increased, which reflected that the rainstorm had moved from the city of Zhengzhou to the city of Xinxiang. On 23 and 24 July in particular, the keywords “rescue”, “repost”, “need”, and “donate” accounted for a large proportion of people’s discussion, implying that the rescue had reached a critical stage and information on the rescue’s progress and the need for relief materials were being widely spread.

Later, from 25 to 27 July, the keywords of “Weihui”, “hard”, and “fighting” continuously took the top spots, indicating that the disaster conditions and rescue had reached quite a difficult situation in Weihui. During these days, the keywords “hero”, “respect”, “grateful”, and “touched” started to appear in people’s discussion, which could be attributed to the spread of touching stories of heroes who participated in the disaster rescue processes.

From 28 July, the public’s topics of concern demonstrated an obvious change in that “responsibility”, “ashamed”, and “government” occupied significant positions due to the official investigation into the Zhengzhou subway incident and the identification of the incident liability. It also raised the public’s sympathy for the victims of this incident, leading to an increase in the frequency of the keywords “RIP” and “sadness”.

#### 3.1.2. The Popularity of Rainstorm-Related Micro-Blogs

**Figure 3:**
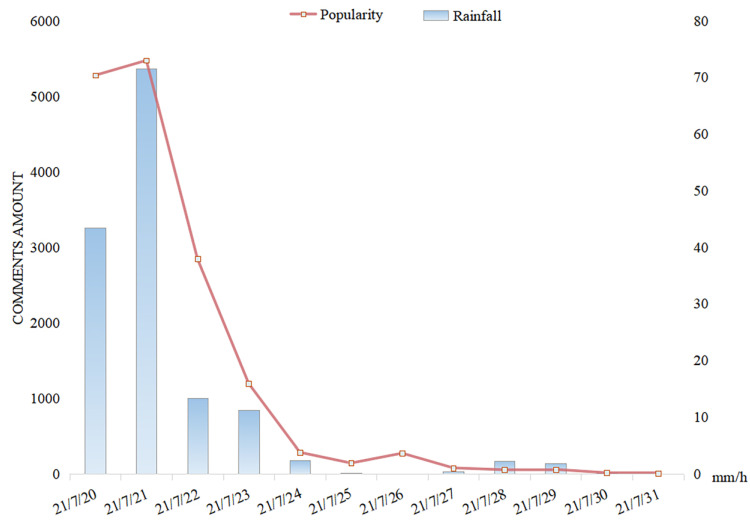
The popularity of related micro-blogs and the precipitation during the rainstorm (daily data).

The daily popularity changes of micro-blogs concerning the rainstorm are shown in Figure 3. The vertical axis measures the total number of comments, indicating the popularity of micro-blogs. The popularity was at a relatively high level on 20 July and reached the highest point on 21 July. After that, the heat of the discussion gradually decreased over time, which was largely consistent with the evolution of the rainfall. Taking into account people’s topics of concern in Figure 2, and that the event-related keywords “Zhengzhou”, “rescue” and “trapped” ranked high on 20 and 21 July, this significant peak was mainly due to the heated discussion of the severe Zhengzhou Metro incident caused by the rainstorm.

**Figure 4:**
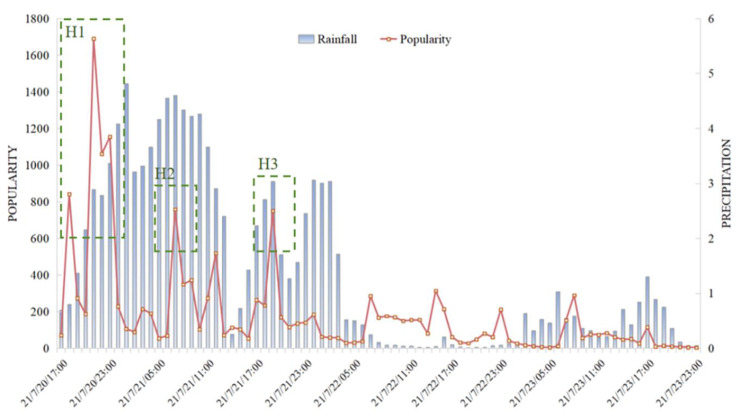
The popularity of related micro-blogs and the precipitation during the rainstorm (hourly data).

In order to further examine the popularity changes when the popularity was at a relatively high level, the number of comments from 20 to 23 July is specifically analyzed in Figure 4 and Table 1. It is shown that the hourly popularity changes did not fit as well with the precipitation as did the daily popularity data, especially for the period of midnight. Here, we used the one-third and two-thirds values of the popularity peak as the criteria of high and medium levels of popularity. Accordingly, three peaks were determined (peaks within two hours were considered as the same peak). Referring to people’s topics of concern in Figure 2, the first and the highest popularity peak, which lasted from 18:00 until 23:00 on 20 July, could be attributed to the Zhengzhou Metro incident, which aroused strong concern and heated discussion on both the situation and the progress of the rescue operation.

On 21 July, several outbreaks of intense discussion occurred, which, referring to people’s topics of concern and the micro-blog content, was the consequence of secondary incidents of the rainstorm. Specifically, the first discussion peak on 21 July appeared at 7:00, which most likely captured the remaining heat of the discussion on the Zhengzhou Metro event of the previous day because the comment keywords during this time period remained almost the same as the day before. The second peak on this day occurred at about 19:00, which was due to the publication of a comic expressing encouragement for the victims in Henan Province.

From 22 July, there were still some small discussion peaks on the rainstorm, while both the length and the strength decreased remarkably compared with the previous peaks. The discussion peaks, according to the micro-blog content, were also the consequences of the secondary incidents of the rainstorm, such as the updated information about the casualties and reports on the suffering.

It is worth mentioning that there were always low ebbs between 0:00 to 6:00 at night, which is consistent with the general public’s timetable for sleeping. As a result, sharp decreases in discussion heat usually occurred at 24:00, and sharp increases occurred at around 7:00, especially when the discussion heat was at a high level the day before.

#### 3.1.3. The Emotion of Micro-Blog Comments

**Figure 5:**
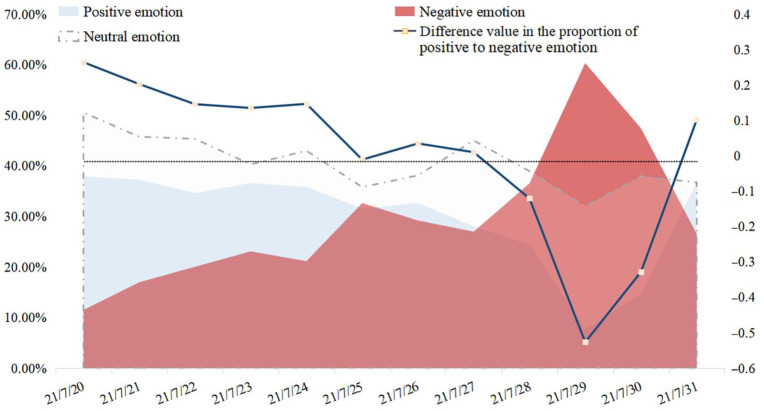
The probability of positive, neutral, and negative comments during the rainstorm (daily data).

The sentiment polarities of people’s micro-blog comments were analyzed, and the daily proportions of positive, negative, and neutral comments are shown in Figure 5. The differences between the positive and negative proportions were calculated to represent the general state of people’s emotion. The larger the difference value, the more positive the public’s emotion. On the whole, the difference value stayed over 0%, indicating that positive comments occupied dominant positions in the early stages before 27 July compared with negative comments. At the beginning of the disaster, when the incident of Zhengzhou Metro happened, the difference value was at its highest point, which could be due to the public’s encouragement for the affected victims, as Figure 2 shows.

On 28 July, the proportion of negative emotion increased rapidly. Later, on 29 July, the proportion of positive comments hit the lowest point, and the negative emotion reached 60% on 29 July. According to the corresponding micro-blog contents and the public’s topics of concern in Figure 2, this might be attributed to the exposure of the statistical results on the casualties of the rainstorm, which triggered the public to reflect on the disaster prevention and emergency measures of the relevant government departments that did not meet the public’s satisfaction. After that, the difference value returned to normal levels on 31 July.

**Figure 6:**
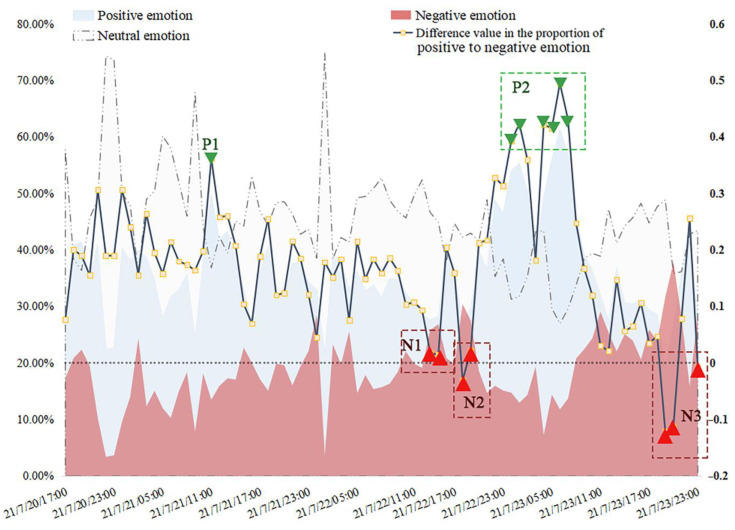
The probability of positive, neutral and negative comments during the rainstorm (hourly data).

As with the analysis of popularity, the hourly emotional polarities were also analyzed. The black line in Figure 6 represents the difference value in the proportions of positive to negative emotions. Here, we used the mid-value of the maximum difference from the mean value of the emotional evaluation results as the criteria for the positive emotion and negative emotion peaks. Finally, two positive peaks and three negative peaks were identified, and the causes of these peaks are listed in Table 2, referring to the public’s topics of interests in Figure 2 and the micro-blog contents at the corresponding times.

### 3.2. The Relationship Between Micro-Blog Popularity and Comment Emotion

In order to further distinguish the relationship between the public’s discussion popularity and their emotion polarity, a correlation analysis was conducted, and the results are shown in Table 3. Here, the discussion popularity was represented by the hourly numbers of micro-blog comments from 20 to 23 July, when the online discussion was most intense, as well as the emotion polarity according to the proportion of positive, negative, and neutral emotions in the same time period. There was a significant correlation between the popularity of the disaster-related micro-blogs and the proportions of negative and neutral emotions. The proportions of negative emotion decreased with the increase in popularity, while the proportion of neutral emotion rose when the discussion popularity grew. In other words, as the discussion heated up, the proportion of negative emotions dropped, and the spaces for neutral and rational speeches grew significantly.

### 3.3. Levels of the Public Opinion Events

**Figure 7:**
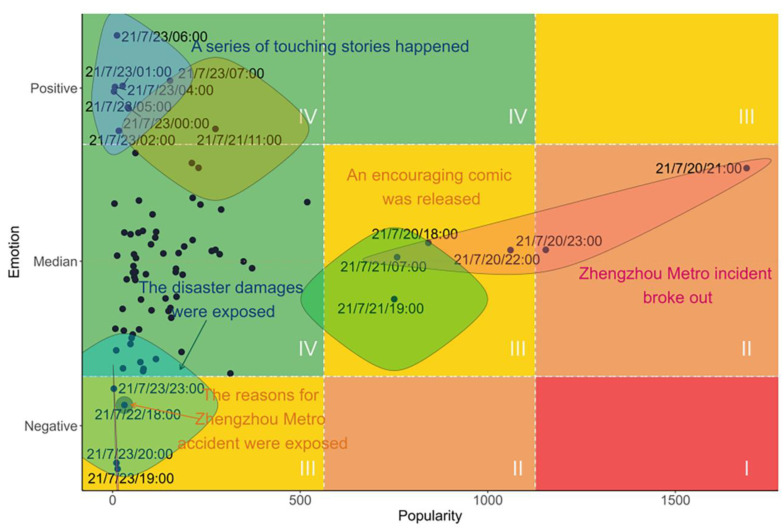
The classification of public opinion events in the case of 7.20 Henan rainstorm.

Using the same popularity and emotion criteria as described in Section 3.1, all the public opinion events could be classified into nine types, which are shown in Figure 7. In the process of public opinion monitoring, events with the characteristics of low or medium popularity and positive or neutral emotion (area IV in Figure 7) are in a normal condition, which do not require attention. On the contrary, conditions with high popularity and negative emotion (area I in Figure 7), which are recognized as negative public opinion events, should be avoided if possible. Therefore, the patterns of this event type need to be recognized in time in order to avoid negative public opinion events and create a favorable environment for public opinion. In the case of the 7.20 Henan rainstorm, it was found that no negative public opinion conditions appeared. However, we can still learn the public opinion patterns from other events that have high popularity or negative emotions.

## 4. Discussion

Through our analysis, several key findings have emerged that contribute to the existing body of knowledge on the causes and characteristics of public opinion events.

First, based on the above analysis, one significant factor contributing to the high popularity of micro-blogs is the abrupt occurrence of disaster events, suggesting that information content is a crucial determinant of popularity. In the daily data, the popularity of disaster-related micro-blogs was highly correlated with the precipitation level, indicating that severe disaster conditions could increase the public’s intense discussion on online social networks, which was consistent with general life experience and previous research findings (Han et al., 2020; Kim et al., 2018; Kim & Hastak, 2018; Naskar et al., 2020). Moreover, the high popularity only occurred when the events were first exposed to the public (the peaks of H1 and H2 in Figure 4). Although similar events in recent days could also raise people’s attention, the discussion heat declined sharply.

Second, information content also contributes to shaping the emotional expression of the public. In general, the public’s emotion remained roughly positive and stable in the long run, even when unexpected events occurred, which is consistent with most previous research findings (Pang et al., 2020). In the stage of post-disaster disposal, the causes of the incidents and the statistical results of disaster damage were investigated, arousing the public’s condemnation for the failures of the responsible departments and persons in handling the situation. In the short term, people’s emotion could strongly fluctuate, being affected by the disaster situation development and the release of related information. Essentially positive emotions were found to be triggered by the moving stories of heroes who participated in the rescue works. On the contrary, situations that triggered the public’s negative emotions included the following: (a) relevant persons and departments not handling the emergency events well, resulting in unfortunate consequences; (b) negative social events that occurred during the disaster; and (c) moments when severe disaster damages were exposed to the public. On the one hand, when the statistical results of the affected regions were announced, the severe casualties and property loss aroused people’s sympathy and sadness. On the other hand, the causes of the secondary accidents that could have been avoided through effective actions were exposed, which increased the public’s complaints and condemnation regarding relevant departments who they felt should be held responsible for the accidents. These results could also be supported by the one-way analysis of variance results indicating that micro-blogs on rescue progress earn the most positive comments and those on disaster disposal receive the most negative comments.

It is worth mentioning that the severe disaster situation itself did not directly cause the public’s negative emotions; it raised their sympathy for the victims and their expressions of wishes and encouragement instead. The main factor that truly resulted in negative public opinion was the relevant departments’ late or improper handling measures. For example, severe disaster damage occurred due to the dereliction of relevant departments and responsible persons, which gave rise to the public’s condemnation (the peak of negative emotion on 29 July in Figure 5; point N2 and N3 in Figure 6). So, the proper and effective handling of disasters and secondary incidents could create a virtuous circle of public opinion, while improper or late handling and information release could tremendously intensify the negative emotion displayed on disaster-related micro-blogs and would be more likely to cause a large public opinion crisis.

Third, the form in which information is conveyed is also a crucial determinant of its popularity. Novel and interesting forms of information could raise the popularity of micro-blogs. For instance, the level of popularity was relatively low in the morning of 21 July, but the introduction of heartwarming and endearing comics sparked a fresh surge in the intensity of discussion (see the H3 peak in Figure 4). Similar results have also been elaborated in previous academic studies; i.e., that highly vivid posts on brand fan pages enhance the interaction between companies and consumers (De Vries et al., 2012; Khan et al., 2016).

Fourth, it was found that proper linguistic cues from official agencies could significantly exert an influence on the emotional inclination of public opinion (M. Cai et al., 2021). In addition to the promotion of positive stories, as mentioned above, positive and encouraging discourse from authoritative agencies could also effectively change public sentiment from negative to positive, as seen in the H1 and H2 peaks in Figure 4. Typically, when a disaster occurs and leads to severe consequences, it is widely believed that it will cause public concern, panic, and skepticism, thereby generating negative public opinion. Yet, in this specific instance, despite the extreme rainstorm and the Zhengzhou Metro incident sparking a high level of discussion, the prevailing emotions expressed were positive. This could be attributed to the media agency’s strategic employment of affirmative language such as “wish”, “safe and peace”, “hold on”, “fighting”, and “get united” during the dissemination of information, and empirical evidence from previous studies has substantiated this observation (Christensen & Lægreid, 2020; Sun et al., 2022).

Lastly, it was found that the proportion of negative comments decreased while that of neutral comments increased significantly with the rise in a micro-blog’s popularity. This showed that the majority of the public were rational and not easily infected by negative emotion. They tended to reflect on both the positive and negative comments and eventually reached a balanced attitude towards the micro-blog contents. This presents a different conclusion from previous studies, which suggested that emotional expressions are closely associated with high popularity (Stieglitz & Dang-Xuan, 2013; X. Wang et al., 2018). In our opinion, the dynamics of popularity and public emotion are closely intertwined with the progression of events. The surge in popularity typically accompanies the emergence of new events, while negative public emotion arises in the wake of man-made adverse incidents, such as the unintended consequences of a government’s inadequate emergency response. Consequently, the distinct origins of popularity and negative emotion can lead to a divergence in the timing of their peaks. Additionally, there has been a notable phenomenon observed within the realm of social media, wherein initially fervent public emotions have been aroused by the exposure of malpractices such as the manipulation by internet propagandists and the misquotation of media content, which collectively contribute to a shift in user behavior. Therefore, the public has transitioned from an eagerness to engage in intense emotional reactions or to disseminate hot-button public opinion narratives to adopting a more circumspect stance, which corroborates the hypothesis posited by prior research that public opinion possesses an inherent “self-purification” function (Z. Wang & Chen, 2021; Y. Zhang et al., 2021).

In summary, our study has delineated that the popularity of disaster-related information and the emotion of public comments cannot be simply attributed to the content or form of the information. Their interplay is multifaceted and intricate. Our findings indicate that the popularity of public discourse is associated with new events and novel forms of information, while the public’s emotional expressions are linked to the nature of the event itself and the linguistic cues provided by the media agencies. The aforementioned findings can be elucidated and supported by past research on novelty preferences and appraisal theory.

In summary, our study has underscored the significant roles of both content and form in shaping the popularity of disaster-related information and the emotional tenor of public comments. The interplay between these two dimensions is multifaceted and intricate. Specifically, our findings reveal that the popularity of public discourse is closely associated with the introduction of contents about new events and novel forms of information presentation. Meanwhile, the emotional expressions of the public are intricately linked to the inherent nature of the event itself, as the linguistic cues provided by media agencies are generally presented through information content. These insights highlight the intertwined influences of content and form on public engagement and emotional response. The aforementioned findings can be elucidated and supported by past research on novelty preferences and appraisal theory.

On the one hand, previous empirical studies reveal that novelty preferences are more dominant in the context of natural scenes compared with the familiarity preference, as seen in both infants and adults favoring novel stimuli over familiar ones (Liao et al., 2011). The mechanism underlying this preference may be linked to the biased competition hypothesis for novelty preference. This hypothesis, as proposed by (Snyder et al., 2008), suggests that novel stimuli inherently elicit attentional biases, capturing attention due to their novelty. Similar research conclusions have been reached within the realms of journalism and media studies. For instance, an analysis of a dataset comprising cover stories was conducted, revealing that novelty is significantly correlated with heightened sales figures (Franceschelli, 2011). Additionally, a study examining the impact of a one-day delay in the reporting of breaking news by newspapers in Argentina demonstrated a substantial 65% decrease in readership, underscoring the critical role of timeliness, which means a certain degree of novelty, in media consumption.

On the other hand, the emotional expressions of the public on social media can be elucidated by the appraisal theory. It originates from the seminal works of Arnold (1960), Arnold (1960) and Lazarus (1966), who initially employed the term “appraisal” in the technical sense. This concept underwent significant evolution during the early 1980s, culminating in the contemporary understanding of appraisal theories (Scherer, 1999; Schorr, 2001). These theories propose that most emotions are elicited and differentiated based on individuals’ evaluations of the events for their well-being (Ellsworth & Scherer, 2003). They view the components of emotion as part of a sequential process, starting with an evaluation of the stimulus as good or bad, pleasant or unpleasant, followed by more complex appraisals (Ellsworth & Scherer, 2003; Scherer, 2009a, 2009b), providing a comprehensive framework for understanding the dynamics of emotional responses. Appraisal theory has been applied across various domains, including the analysis of the public’s emotional expression on social media. Empirical studies have shown that specific appraisal dimensions in social media posts elicit discrete emotions, such as anger or sadness, demonstrating the practical application of appraisal theory in understanding emotional responses in social media contexts (Zerback & Wirz, 2021). Based on appraisal theory, this study posits that the positive or negative nature of disaster-related information posted on social media, along with the positive or negative emotions conveyed by the information publisher, provide the public with either positive or negative stimuli. These stimuli trigger corresponding appraisal processes, which manifest in the emotional polarization of public comments.

## 5. Conclusions

It is critical for the government to understand the evolution and formation mechanisms of public opinion during disasters so as to prevent a severe public opinion crisis as far as possible. In order to examine the public opinion expressed on online social networks during disasters, this paper, drawing on previous research, proposed an analytical framework for analyzing public opinion during disasters by considering aspects such as the public’s topics of concern, topic popularity, and comment emotions. This framework emphasizes the effect of media agencies on shaping the public opinion environment, clarifies the relationship among the three factors of public opinion, and proposes a new perspective from which to evaluate public opinion events, which could provide a possible direction for the in-depth analysis of public opinion.

Guided by this framework, we analyzed the popularity of disaster-related micro-blogs and the emotional tendency of the micro-blog comments using the case of the Henan rainstorm on 20 July 2021. Our analysis yielded several significant findings. First, the topics of concern, topic popularity, and emotional tendency of the public were found to be largely consistent with the evolution of the disaster, particularly when observed through daily data. Additionally, the hourly data revealed a marked susceptibility to diurnal human activity patterns, with a pronounced decline in topic popularity at midnight. Secondly, our study indicates that the popularity of disaster-related information and the emotion of public comments on social media are associated with the content and form of the information. Novel informational content or innovative forms of information contribute to enhancing the popularity of topics, while the events or emotions expressed within the information elicit corresponding emotional responses from the public, which are corroborated by previous studies on novelty preference and appraisal theory. These conclusions are conducive to assisting governmental authorities in refining the public opinion environment and averting the occurrence of adverse public opinion incidents. Third, our analysis has identified a pronounced correlation between the popularity of disaster-related micro-blogs and the distribution of negative and neutral emotions within the discourse. Notably, as popularity increased, the prevalence of negative emotions was observed to diminish concurrently with a rise in the proportion of neutral emotions.

The findings above revealed the causes leading to negative public emotion events with high popularity and negative public emotions. Generally, both the popularity and public emotion evolve with the disasters, so they are associated with each other. While the causes of high popularity and negative emotion are different, determining that their occurrence is not always temporally congruent, which accounts for the observed association of heightened popularity with positive emotions in certain instances and with negative emotions in others. Furthermore, the increasingly cautious attitude with which the public engages with public opinion has engendered a self-purification function within the discourse, leading to a prevalence of neutral emotions in the public emotion landscape. These findings highlight the intricate interplay between the dissemination of information and the evolution of public emotion, suggesting a complex and adaptive mechanism within the social media ecosystem. These findings extend and deepen the current academic understanding of this topic, providing a novel lens through which future scholarly research may be conducted. It underscores the complexity of public opinion dynamics and the adaptive nature of social media interactions, suggesting that the interplay between information dissemination and emotional response is a multifaceted process that warrants further exploration and analysis.

According to the above results and discussion, some suggestions for the management of public opinion were put forward, as follows:

- Plan the key points of public opinion management according to time.

On the one hand, it was found that high popularity usually occurs at the very beginning of an unexpected event. So, the monitoring of public opinion in terms of frequency and intensity should be strengthened in this period (Su et al., 2021). On the other hand, considering that the popularity of micro-blogs usually drops to a significantly low level at night, midnight could be an excellent time for relevant departments to make a detailed and thorough consideration of the coping strategies for public opinion, i.e., with no need to worry about the introduction of new, unexpected conditions. Unfavorable information could be controlled, and some positive information could also be released at midnight to reverse the passive situation. On the contrary, the development of public opinion should be paid close attention during the daytime when the public are relatively active.

- Handle the initial disaster and secondary accidents effectively and in a timely manner.

It was found that the disaster itself would not raise the public’s dissatisfaction and complaints, but the failures of relevant departments in preventing and controlling the disaster’s consequences were likely to stimulate the public’s negative emotions, especially in cases when severe casualties occurred. Therefore, controlling disaster consequences in time in an effective manner is a fundamental measure for the prevention of an adverse public opinion crisis (B. Zhang et al., 2022). Possible approaches include improving the emergency response capacity of emergency management authorities and the public, improving disaster and emergency plans, strengthening the monitoring and early warning of secondary disaster events, and so on.

- Organize the contents of disaster-related micro-blogs carefully and reasonably.

According to our research findings, varying micro-blog contents could lead to different expressions of public opinion. To maintain a positive public opinion environment, more positive information, such as touching stories and news of the satisfactory progress of the rescue works, could be released in attractive ways (B. Zhang et al., 2022). As to information with negative content, like the post-disaster disposal, feasible measures to reduce the public’s negative emotion include appending some positive information, like the efforts of the relevant departments to prepare for and handle future disaster, proactively admitting the mistakes and putting forward corrective actions.

Our study comes with limitations, particularly due to the exclusive focus on data from Sina Weibo. This singular platform selection may not fully capture the diverse patterns and characteristics of public opinion evolution across different online social networks. For instance, previous research has shown that public concerns and user interactions vary significantly between platforms such as Weibo, Twitter, and Facebook (Deng & Yang, 2021; Y. Yang et al., 2021), leading to distinct focuses and emotional expressions. Therefore, extending our analysis to multiple platforms could provide a more comprehensive understanding of public opinion dynamics during disasters. Moreover, this research used only one rainstorm examples to study public opinion during disasters. Analyzing more disaster examples in similar investigations could assist researchers in learning about the changes in public opinion more accurately and fully through qualitative and quantitative measures.

Additionally, our study did not account for potential confounding factors that might influence information popularity and public emotion. Factors such as user demographics, network characteristics, and the credibility of information sources could all play significant roles in shaping public responses. Future research could benefit from exploring these confounding factors to provide a better understanding of public opinion.

Furthermore, our study did not explore the longitudinal evolution of public opinion over extended periods post-disaster. Future work could incorporate longitudinal analyses to provide deeper insights into the temporal dynamics of public opinion following disaster events.

## Figures and Tables

**Table 1:** Information about the popularity peaks from 20 to 23 July.

Peaks	Time	Comments’ Keywords	Events	Communication Strategy
H1	18:00 to 23:00, 20 July	“Zhengzhou”, “trapped”, “water”, “rescue”	Zhengzhou Metro incident broke out, and the rescue works were underway.	“wish”, “safe and peace”, “rescue”, “take care”, “urgent spread”
H2	7:00, 21 July	“Zhengzhou”, “rescue”, “fighting”	Zhengzhou Metro incident broke out, and the rescue works were underway.	“rescue”, “hold on”, “fighting”, “get united”
H3	19:00, 21 July	“fighting”, “wish”, “be careful”	People from other provinces expressed their encouragement for the victims in Henan Province.	Use the form of comics to express the warm encouragement to the victims from all over the country.

**Table 2:** Information about the emotion peaks in Figure 6.

Emotion Types	Peaks	Time	Comments’ Keywords	Events	Communication Strategy
Positive	P1	11:00, 21 July	“rescue”, “respect”, “grateful”	A series of touching and positive stories that happened during the disaster relief progress were widely spread.	“safe and peaceful”, “ thanks”, “rescue”
	P2	0:00 to 5:00, 23 July	“rescue”, “respect”, “hero”	A series of touching and positive stories that happened during the disaster relief progress were widely spread.	“hero”, “salute”, “rescue”
Negative	N1	14:00 to 15:00, 22 July	“sadness”, “rescue”, “RIP”	A public official died during rescue, raising the public’s sadness and regrets.	“swept away by floodwaters when rescuing people”
	N2	18:00 to 19:00, 22 July	“sadness”, “metro”, “Zhengzhou”, “reason”, “responsibility”	The reasons for the Zhengzhou Metro accident were exposed and the related departments received the public’s censure.	“More than 500 passengers were trapped”, “twelve passengers died”
	N3	19:00 to 23:00, 23 July	“sadness”, “RIP”, “Xinxiang”, “hard”, “need”	The statistical results of the disaster damage were exposed, increasing the public’s sadness and regrets.	“51 people died”, “urgent evacuation”, “crop damage”, “economic losses”

**Table 3:** Correlation analysis between topic popularity and comment emotions.

		Topic Popularity
Positive emotion	Pearson correlation	−0.163
	Significance (bilateral)	0.151
Negative emotion	Pearson correlation	−0.341 **
	Significance (bilateral)	0.002
Neutral emotion	Pearson correlation	0.393 **
	Significance (bilateral)	0.000

Note: ** *p* < 0.01.

## Data Availability

The data presented in this study are available on request from the first author.
